# A Novel Calcium‐Ion Battery Based on Dual‐Carbon Configuration with High Working Voltage and Long Cycling Life

**DOI:** 10.1002/advs.201701082

**Published:** 2018-04-27

**Authors:** Shi Wu, Fan Zhang, Yongbing Tang

**Affiliations:** ^1^ Functional Thin Films Research Center Shenzhen Institutes of Advanced Technology Chinese Academy of Sciences Shenzhen 518055 China; ^2^ Nano Science and Technology Institute University of Science and Technology of China Suzhou 215123 China

**Keywords:** calcium‐ion battery, dual‐carbon, graphite cathode, intercalation

## Abstract

Rechargeable batteries based on multivalent cations (e.g., Mg^2+^ and Al^3+^) have attracted increased interest in recent years because of the merits of natural abundance, low cost, good chemical safety, and larger capacity. Among these batteries, the Ca‐ion battery (CIB) shows attractive priority because Ca^2+^ has the closest reduction potential (−2.87 V vs standard hydrogen electrode (SHE)), to that of Li (−3.04 V vs SHE), enabling a wide voltage window for the full battery. However, most Ca‐ion batteries have low working voltage (below 2 V), as well as poor cycling stability (less than 50 cycles). Here, a high‐performance Ca‐ion full battery with a novel dual‐carbon configuration design with low‐cost and environmentally friendly mesocarbon microbeads and expanded graphite as the anode and cathode, respectively, is reported. This Ca‐ion‐based dual‐carbon battery (Ca‐DCB) can work successfully in conventional carbonate electrolyte dissolving Ca(PF_6_)_2_, with a reversible discharge capacity of 66 mAh g^−1^ at a current rate of 2 C and a high working voltage of 4.6 V. Moreover, the Ca‐DCB exhibits good cycling stability with a discharge capacity of 62 mAh g^−1^ after 300 cycles with a high capacity retention of 94%, which is the best performance of the reported CIBs, suggesting it is a promising candidate for next‐generation energy storage devices.

Rechargeable batteries based on multivalent cations (e.g., Mg^2+^,^1^, ^2^ Al^3+^,^3^ Zn^2+^,^4^) have attracted increased interest in recent years. Except for the merits of high natural abundance, low cost, and good chemical safety compared to lithium‐ion batteries, one outstanding property is that a multivalent charged ion can accept more electrons for one single ion than the monovalent ions such as Li^+^, Na^+^, and K^+^, contributing to larger capacity under the same concentration.^5^ Among the multivalent‐cation‐based batteries, Ca‐ion battery shows attractive priority as Ca^2+^ has the closest reduction potential (−2.87 V vs standard hydrogen electrode (SHE)) to that of Li (−3.04 V vs SHE), enabling wide voltage window for the full battery.^5^, ^6^, ^7^, ^8^ In addition, Ca^2+^ has faster electrochemical kinetics than Mg^2+^ due to its lower polarizing property,^6^ thus leading to better rate performance. Ca^2+^ has been found to be reversibly intercalated into host materials such as V_2_O_5_ 9, 10 and Prussian blue analogs.^5^, ^11^ However, the development of a Ca‐ion battery is sluggish due to the slow diffusion of Ca^2+^ into the active materials, and most reported Ca‐ion batteries exhibited low working voltage (<2.0 V), as well as poor cycling stability (within 100 cycles).^5^, ^9^, ^10^, ^11^ Moreover, when calcium metal is used as the anode material, electrolytes would be confined to acetonitrile‐based electrolytes for good stability, while corrosion would occur in carbonate‐based electrolytes.^10^ Aurbach et al. pioneered the work of Ca‐ion battery and discovered that Ca metal electrode in conventional organic electrolytes is apt to form surface passivation film, which prevents Ca^2+^ transportation thus leading to irreversible calcium deposition.[[qv: 7a]] Although some researchers realized reversible Ca deposition in molten electrolyte, the working temperature is extremely high (550–700 °C),[[qv: 7b]] which cannot match the mainstream application conditions.

Replacing the metallic calcium anode by intercalation‐type active material is a feasible strategy to avoid calcium plating and stripping. To address the above issue, herein, we first report a new configuration of Ca‐ion full battery with low‐cost and environmentally friendly carbon resources as anode and cathode materials. This designed Ca‐ion‐based dual‐carbon battery (denoted as Ca‐DCB) can work reversibly at room temperature in conventional carbonate electrolyte dissolving Ca(PF_6_)_2_, with a discharge capacity of 66 mAh g^−1^ at a current rate of 2 C at an extremely high working voltage up to 4.6 V. Moreover, this Ca‐DCB exhibited a good cycling stability with a discharge capacity of 62 mAh g^−1^ after 300 cycles with a high capacity retention of 94%, which is the best performance of the reported Ca‐ion batteries.

The battery configuration and the charging/discharging process of the designed Ca‐DCB are schematically shown in **Figure** **1**a. The mesocarbon microbead (MCMB) was utilized as anode material in the Ca‐ion battery for the first time, which has isotropic graphitic layered structure, thus is beneficial for ion intercalation/deintercalation, especially for the large Ca^2+^.^12^ The working mechanism illustrated in Figure 1a is discussed as follows. During charging, Ca^2+^ ions move to the MCMB anode and intercalate into the graphite layers forming CaC*_x_* intercalation compound, and at the same time PF_6_^−^ anions move to the expanded graphite (EG) cathode and intercalate into the graphite layers forming C*_y_*(PF_6_) intercalation compound. Typical galvanostatic charge/discharge curves of the Ca‐DCB are shown in Figure 1b. The working voltage window was set to be 3.0–5.2 V owing to high‐potential plateaus of the Ca‐DCB. It can be clearly seen that the charging curve can be divided into three regions between 4.10 – 4.85 V (stage I), 4.85 – 4.99 V (stage II), and 4.99 – 5.20 V (stage III), which are corresponding to different stages of PF_6_^−^ intercalation into EG cathode. During discharging, three regions were also found in the discharge curve with 5.20 – 4.65 V (stage III′), 4.65 – 4.25 V (stage II′), and 4.25 – 3.50 V (stage I′), which represent different stages of PF_6_^−^ deintercalation from EG cathode. The charge/discharge curves shown in Figure 1b are very similar to the lithium‐ion‐based dual‐carbon batteries,^13^ owing to the close reduction potential of Ca^2+^ and Li^+^ (only 0.17 V).^6^ The corresponding d*Q*/d*V* differential curves shown in Figure 1c further revealed three charge plateaus and three discharge plateaus of the Ca‐DCB during charge/discharge process at 2 C, respectively. As the Ca‐DCB shows a high average discharge voltage of ≈4.6 V, it can easily light up two light‐emitting diodes (LEDs) with red and blue color in series (see the inset of Figure 1c).

**Figure 1:**
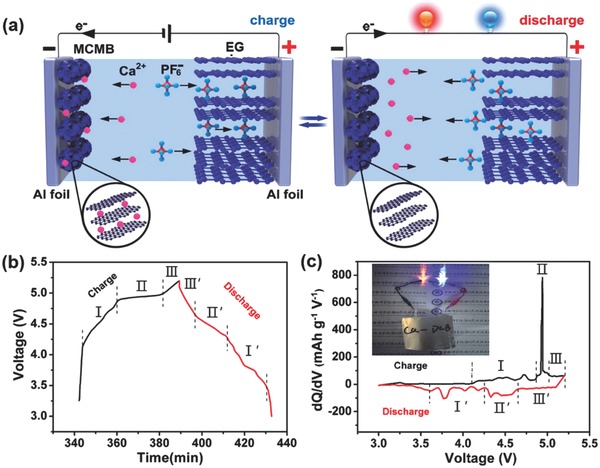
a) Schematic of the configuration of the designed Ca‐DCB battery, which consists of expanded graphite cathode and MCMB anode, with 0.7 m Ca(PF_6_)_2_ in EC/DMC/EMC (4:3:2 v/v/v) as the electrolyte. b) Typical charge/discharge curves of the Ca‐DCB at 1 C. c) Corresponding d*Q*/d*V* differential curves of the Ca‐DCB. Inset is a Ca‐DCB pouch cell which can light up two LEDs with red and blue color in series.

To further illustrate the working mechanism of the Ca‐DCB, characterization on the EG cathode and MCMB anode was performed. According to the charge/discharge curves of the Ca‐DCB (**Figure** **2**a), we chose seven different charging/discharging states for analyzation. Figure 2b shows the ex situ X‐ray diffraction (XRD) profiles of the EG cathode in the Ca‐DCB at different charging/discharging states during the initial cycle. Before charging, the EG cathode shows a sharp (002) diffraction peak at 26.6° which implies high degree of graphitization. During charging, the intensity of the (002) peak greatly decreased and shifted to a lower value, accompanied with a new (00*n*+1) peak at around 25.4° appeared, indicating intercalation of PF_6_^−^ anions into the graphitic layer.[[qv: 3b,14]] With the increase of charging voltage, the intensity ratio of the (00*n*+1) and (002) peaks gradually increased with the shift of the (00*n*+1) peak to lower 2θ value, demonstrating more PF_6_^−^ ions intercalation. During discharging, the (00*n*+1) peak gradually shifted back to higher degree accompanied with the intensity increase of the (002) peak. At fully discharged state, the two peaks merged into one peak at 26.0°, implying PF_6_^−^ ions' deintercalation. However, the intensity of the peak did not recover to the initial state, indicating little irreversibility of the PF_6_^−^ intercalation/deintercalation process.^15^ Ex situ Raman spectra of the EG cathode in the Ca‐DCB at different charging/discharging states during the initial cycle were also observed (as shown in Figure 2c), and similar results were obtained. Before charging, a G‐band peak was observed at 1582 cm^−1^ that corresponds to the E_2g_ vibrational mode originated from the bond stretching of sp^2^ atoms. During charging, the G‐band peak splits into two peaks which were recognized as E_2g2_(i) mode at 1584 cm^−1^ and E_2g2_(b) mode at 1604 cm^−1^, respectively. Furthermore, the E_2g2_(i) peak gradually blueshifted and the intensity ratio of the E_2g2_(b) and E_2g2_(i) peaks increased, corresponding to the PF_6_^−^ anions' intercalation process.[[qv: 3b]] During discharging, the E_2g2_(i) peak gradually redshifted to the original location, and the split peaks gradually merged into one peak, corresponding to the PF_6_^−^ anions' deintercalation process.

**Figure 2:**
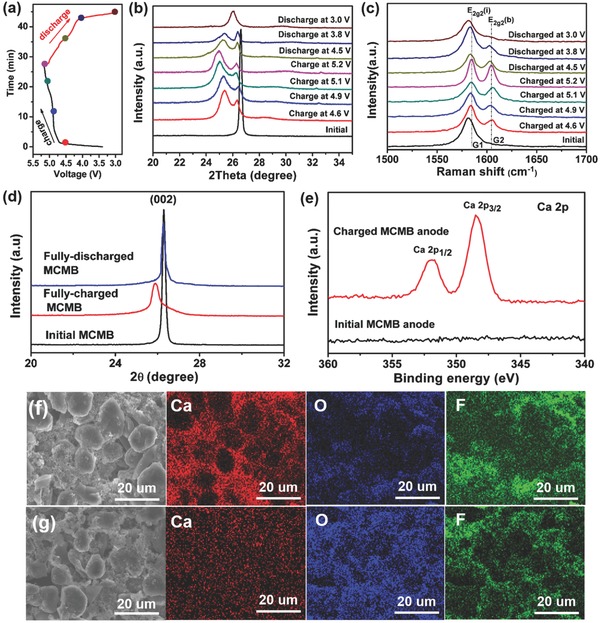
a) Charge–discharge voltage profiles of the Ca‐DCB at 2 C. b) XRD profiles and c) Raman spectra of the EG cathode in the Ca‐DCB at different charging/discharging states during the initial cycle. d) XRD patterns of initial, fully charged, and fully discharged MCMB anode in the Ca‐DCB during the initial cycle. e) XPS Ca 2p spectra of the initial and the fully charged MCMB anode in a Ca‐DCB. f,g) SEM and corresponding EDX mapping images of the MCMB anode in the Ca‐DCB at f) charging and g) discharging state during the 20th cycle.

To investigate the Ca intercalation/deintercalation in the MCMB anode, we further tested the ex situ XRD, X‐ray photoelectron spectroscopy (XPS), and energy dispersive X‐ray (EDX) of the fully charged MCMB anode in the Ca‐DCB, as shown in Figure 2d–g. The XRD pattern of the initial MCMB anode (Figure 2d) clearly shows a sharp and intense (002) diffraction peak at 26.3°, with the *d*_002_ value of 0.339 nm. When the Ca‐DCB was fully charged to 5.2 V in the initial cycle, the intensity of the (002) peak of the MCMB anode obviously decreased, which also shifted to 25.9° and the *d*_002_ value enlarged to 0.344 nm, demonstrating Ca intercalation into the graphitic layer in the MCMB, similar to the phenomenon of *K* intercalation into graphite.^16^ At the fully discharged state, the 2θ degree and the intensity of the (002) peak recover to the initial state, indicating the reversible Ca deintercalation from the MCMB anode. In addition, the XPS data (Figure 2e) clearly show the Ca 2p peaks at 348.4 and 352.0 eV after charging, also indicating the Ca insertion into the MCMB anode. Figure 2f,g shows the EDX mapping images of the full charged/discharged MCMB anode, where Ca element signal can be clearly observed at fully charged state (Figure 2f) with a mass ratio of 2.04% (Table S1, Supporting Information), thus also indicating the intercalation of Ca into the graphitic layer of MCMB. At the fully discharged state, the Ca content decreased to 0.4%, indicating the deintercalation of Ca^2+^ with little irreversiblity. It is also noted that the contents of O and F on the surface of both charged (8.94% and 13.75%) and discharged (8.78% and 12.77%) MCMB electrodes are higher than those in the initial state (Table S1, Supporting Information), implying the formation of protective solid electrolyte interface (SEI) film on the surface of the MCMB electrode during charging/discharging.[[qv: 15a]]

Before investigating the electrochemical performance of the Ca‐DCB, electrolyte was optimized for pursuing the best performance. The solvent of the Ca‐ion electrolyte was chosen to be a mixture of ethylene carbonate (EC), dimethyl carbonate (DMC) and ethyl methyl carbonate (EMC), which exhibited superior performance than other compositions and has been widely used in commercial lithium‐ion batteries. We also found that higher amount of EC is beneficial for improved solubility of Ca(PF_6_)_2_ with higher capacity which also facilitates the formation of SEI layer.^17^ However, much more EC content can also decrease the capacity due to the fact that it would bind with PF_6_^−^ anions and prevent them from intercalating into graphite.^18^ As shown in Figure S1 (Supporting Information), the Ca‐DCB based on 0.7 m Ca(PF_6_)_2_ dissolved in EC/DMC/EMC (4:3:2) delivered the highest discharge capacity of 66 mAh g^−1^ compared to 0.6 m Ca(PF_6_)_2_ in EC/DMC/EMC (1:1:1) (51 mAh g^−1^) and 0.8 m Ca(PF_6_)_2_ in EC/DMC/EMC (5:3:2) (60 mAh g^−1^).

The electrochemical performance of the Ca‐DCB based on the optimized electrolyte 0.7 m Ca(PF_6_)_2_ in EC/DMC/EMC (4:3:2) was then characterized. **Figure** **3**a shows typical charge/discharge curves of the Ca‐DCB under various current rates (2, 3, 4, and 5 C) in the voltage window of 3.0–5.2 V. With the increase of the current rate, the charge/discharge curves are similar with little separation of voltage plateau, implying little electrode polarization of the Ca‐DCB. Figure 3b further clearly shows that the reversible discharge capacities of the Ca‐DCB at 2, 3, 4, and 5 C are 66, 64, 61, and 55 mAh g^−1^, respectively, and the corresponding Coulombic efficiency (CE) are 82%, 89%, 92%, and 94% respectively, demonstrating excellent rate performance of the Ca‐DCB. In addition, the capacities recovered to the initial values when the current rate decreased, indicating good electrochemical reversibility of the Ca‐DCB. Moreover, the Ca‐DCB exhibited good cycling stability, which kept stable after 300 cycles with a discharge capacity of 62 mAh g^−1^ at 1 C with 94% retention (Figure 3c), much better than other reported Ca‐ion batteries.^5^, ^6^, ^7^, ^8^, ^9^, ^10^, ^11^ It is noted that the CE of the Ca‐DCB at low current rate was below 90%, which was probably owing to the decomposition of the electrolyte at high working voltage (≈4.6 V), as well as the continuous formation of SEI films on the EG cathode and MCMB anode.^15^ The existence of the SEI films was demonstrated by EDX mapping results (Figures S2 and S3, Supporting Information), which clearly showed the existence of the composing elements C, O, and F on the surface. The CE can be improved by using high‐voltage electrolytes such as ionic liquid,^19^ which will be studied in future work.

**Figure 3:**
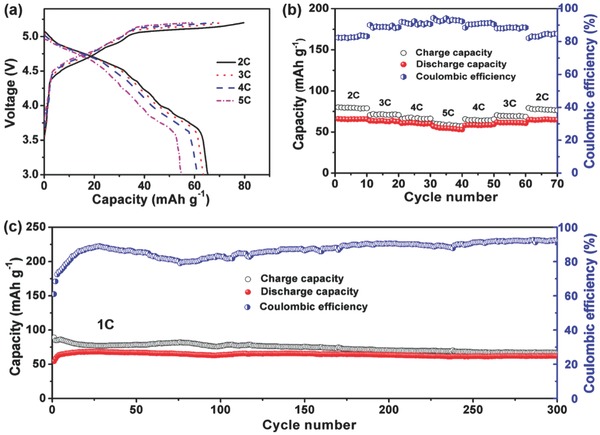
Electrochemical performance of the Ca‐DCB based on 0.7 m Ca(PF_6_)_2_ in EC/DMC/EMC (4:3:2 v/v/v). a) Charge/discharge curves of the Ca‐DCB at various current rates from 2 to 5 C. b) Corresponding charge/discharge capacities and Coulombic efficiency of the Ca‐DCB at different current rates. c) Cycling performance of the Ca‐DCB for 300 cycles at 1 C.

**Figure** **4**a shows the charge/discharge curves of the Ca‐DCB after different cycles. The curves after long cycles did not change much compared to the initial ones, demonstrating good cycling stability of the Ca‐DCB. In addition, the scanning electron microscope (SEM) images of the EG cathode and MCMB anode after 300 cycles (Figures S4 and S5, Supporting Information) show no obvious change, and the XRD patterns of the electrodes after long cycles neither show obvious peak variation except for the depressed intensity of the (002) peak (Figures S6 and S7, Supporting Information), demonstrating good structural stability of the electrodes. Electrochemical impedance spectroscopy measurement was also performed, as shown in Figure 4b. Nyquist plots of the Ca‐DCB after 1, 10, 30, and 50 cycles all show one depressed semicircle corresponding to charge transfer resistance (*R*_ct_) and a sloping line corresponding to Warburg impedance.^20^ The increase of the *R*_ct_ value after ten charge/discharge cycles is mainly due to the SEI film formation on the EG cathode and MCMB anode.^21^ However, the *R*_ct_ value remained almost unchanged after 30 and 50 cycles, indicating that the SEI film on the surface of the electrodes is stable. Moreover, the working voltage (which means the medium discharge voltage) of the Ca‐DCB is as high as 4.6 V and remained very stable for 300 cycles (Figure 4c), also demonstrating good cycling stability.

**Figure 4:**
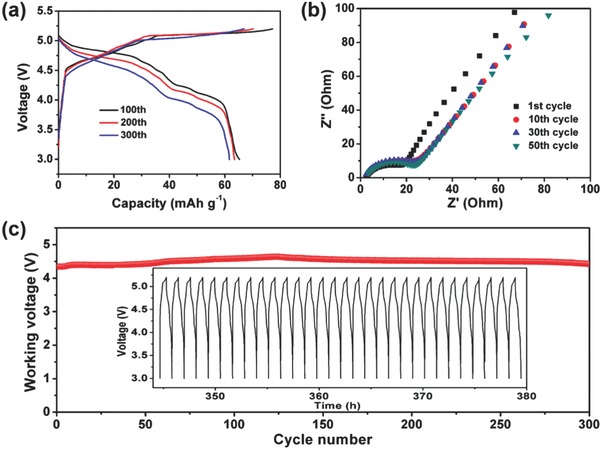
a) Charge/discharge curves of the Ca‐DCB at the 100th, 200th, and 300th cycle at a current rate of 1 C. b) Nyquist plots of the Ca‐DCB after 1, 10, 30, and 50 cycles. c) Working voltage of the Ca‐DCB at 1 C for 300 cycles. Inset is the charge/discharge curves of the Ca‐DCB during the last 30 cycles.

In summary, we have successfully designed a novel Ca‐ion battery with both good rate performance and cycling stability by employing reversible dual graphitic carbon intercalation chemistry with simultaneous accommodation of Ca^2+^ and PF_6_^−^ in MCMB anode and EG cathode, respectively. By using optimized electrolyte, the as‐prepared Ca‐DCB achieved a reversible discharge capacity of 62 mAh g^−1^ after 300 cycles at a current rate of 1 C with 94% retention, much better than previously reported Ca‐ion batteries. Furthermore, the working voltage of the Ca‐DCB is as high as 4.6 V, which is beneficial for enhanced energy density and can satisfy the requirement for high‐voltage devices. With the above performances as well as the merits of large abundance of Ca, low cost and environmentally friendliness, this Ca‐DCB shows potential applications in large‐scale intermittent renewable energy storage fields such as wind and solar energy.

## Conflict of Interest

The authors declare no conflict of interest.

## Supporting information

Supplementary
