# Supplementary material for: A Novel Calcium‐Ion Battery Based on Dual‐Carbon Configuration with High Working Voltage and Long Cycling Life
Source: Adv Sci (Weinh). 2018 Apr 27;5(8):1701082. doi: 10.1002/advs.201701082 (PMC6097003; doi:10.1002/advs.201701082)
Supplement: Supplementary file 1 — Supplementary [file ADVS-5-1701082-s001.pdf]

Copyright WILEY-VCH Verlag GmbH & Co. KGaA, 69469 Weinheim, Germany, 2018.

ADVANCED SCIENCE
Open Access

Supporting Information

for *Adv. Sci.,* DOI: 10.1002/advs.201701082

A Novel Calcium-Ion Battery Based on Dual-Carbon Configuration with High Working Voltage and Long Cycling Life

*Shi Wu, Fan Zhang, and Yongbing Tang**

Supporting Information for

A Novel Calcium-Ion Battery Based on Dual-Carbon Configuration with High Working Voltage and Long-Cycling Life

Shi Wu,† Fan Zhang,† and Yongbing Tang*

S. Wu, Dr. F. Zhang, Prof. Y. B. Tang
Functional Thin Films Research Center, Shenzhen Institutes of Advanced Technology, Chinese Academy of Sciences, Shenzhen, 518055, China.
Email: tangyb@siat.ac.cn

S. Wu
Nano Science and Technology Institute, University of Science and Technology of China, Suzhou, 215123, China.

†S. Wu and F. Zhang contributed equally to this work.

Keywords: calcium-ion battery, dual-carbon, graphite cathode, intercalation

Materials and Methods

Materials. Expanded graphite (EG), mesocarbon microbead (MCMB), acetylene black, polyvinylidene fluoride (PVDF), and N-methyl-2-pyrrolidone (NMP) were purchased from Shenzhen Kejingstar Technology Ltd. Glass fiber sheets (Whatman, 47mm) were purchased from Shanghai Huanao Technology Co. Ltd for using as the battery separator. The components of the electrolyte in Ca-DCB, , including $Ca(PF_6)_2$, ethylene carbonate (EC), diethyl carbonate (EMC), and dimethyl carbonate (DMC) were purchased from Duoduo Chemical Technology Co. Ltd. All the raw materials were used directly without further purification.

Characterization. Ex-situ X-ray diffraction (XRD) analyses of the electrodes were performed on a Rigaku diffractometer operated at 40 kV and 15 mA using Cu K_α radiation (20 kV, λ = 0.154056 nm). Ex-situ Raman spectra of the EG cathode at different charging/discharging states were analyzed by a LabRAM HR Raman spectrometer (532 nm) in the frequency range from 1500 to 1700 cm^{-1}. Field-emission scanning electron microscope (FE-SEM, HITACHI S-4800) was used to analyze the morphology of the electrodes. All the electrodes for XRD, Raman and SEM tests were washed with DMC thoroughly to remove surface adsorbed electrolyte and sealed in an Ar-filled glove box for the prevention of oxidation.

Electrochemical Characterization. The electrochemical performance of the Ca-DCB were measured by using CR2032 coin-type cells. For preparing EG cathode, 80 wt% EG, 10 wt% of conductive carbon black, and 10 wt% of PVDF as binder were mixed with some NMP solvent together to form a homogeneous slurry. The slurry was

coated onto Al foil and dried at 80 °C in vacuum for 12 h. The electrode sheet was then pressed and punched into circular sheets. The MCMB anode was also prepared by a similar way. The cathode and anode circular sheet are 10 and 12 mm in diameter respectively with mass loading of ~1.0 and ~2.0 mg cm^{-2} respectively. Glass fiber was used as the separator by cutting into circular sheets with 16 mm in diameter and vacuum dried at 120 °C for 10 h. The optimized electrolyte of the Ca-DCB was 0.7 M $Ca(PF_6)_2$/EC-DMC-EMC (4:3:2 v/v/v). The coin-type battery were fabricated in a glove box (Etelux Lab2000) filled with argon. Battery test system (LAND CT2001A) was used to carry out the galvanostatic charge-discharge measurements of the Ca-DCB. Electrochemical impedance spectroscopy (EIS) measurements were performed on an Autolab (PGSTAT302N, Switzerland) electrochemical workstation. The capacities of the Ca-DCB were calculated based on the mass of the cathode material.

Figures and Tables

Table S1. EDX elemental mapping results of the surface of the initial MCMB anode, charged and discharged MCMB anode during the 20th cycle in the Ca-DCB.

**Initial MCMB anode**	**Element**	**Ca**	**C**	**O**	**F**
	**Mass ratio (%)**	0	88.21	2.51	9.28
**Charged MCMB anode**	**Element**	**Ca**	**C**	**O**	**F**
	**Mass ratio (%)**	2.04	75.27	8.94	13.75
**Discharged MCMB anode**	**Element**	**Ca**	**C**	**O**	**F**
	**Mass ratio (%)**	0.4	78.05	8.78	12.77

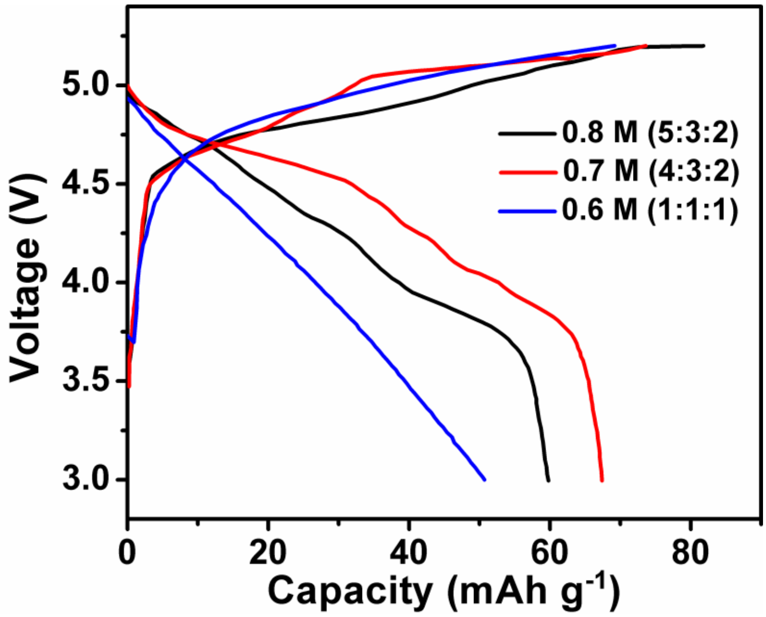

Figure S1. Charge/discharge curves of the Ca-DCB in different electrolytes including 0.8 M $Ca(PF_6)_2$ in EC/DMC/EMC (5:3:2 v/v/v), 0.7 M $Ca(PF_6)_2$ in EC/DMC/EMC (4:3:2 v/v/v), and 0.6 M $Ca(PF_6)_2$ in EC/DMC/EMC (1:1:1 v/v/v).

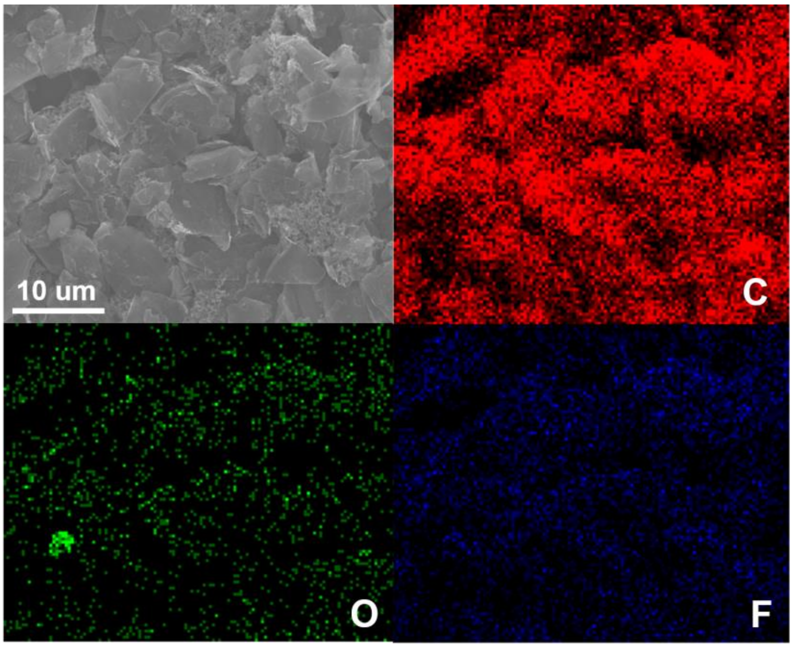

Figure S2. EDX mapping images of the surface of the EG cathode in the Ca-DCB after 300 cycles.

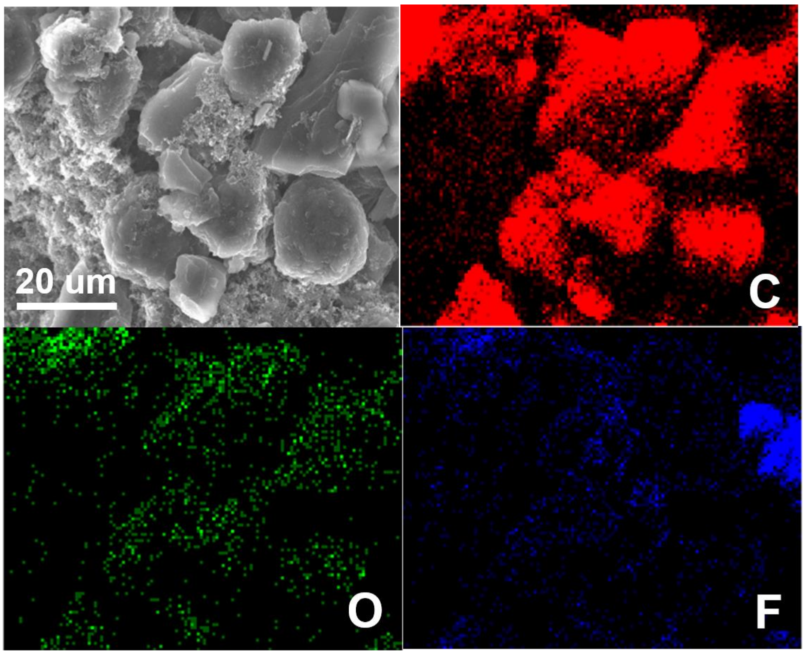

Figure S3. EDX mapping images of the surface of the MCMB anode in the Ca-DCB after 300 cycles.

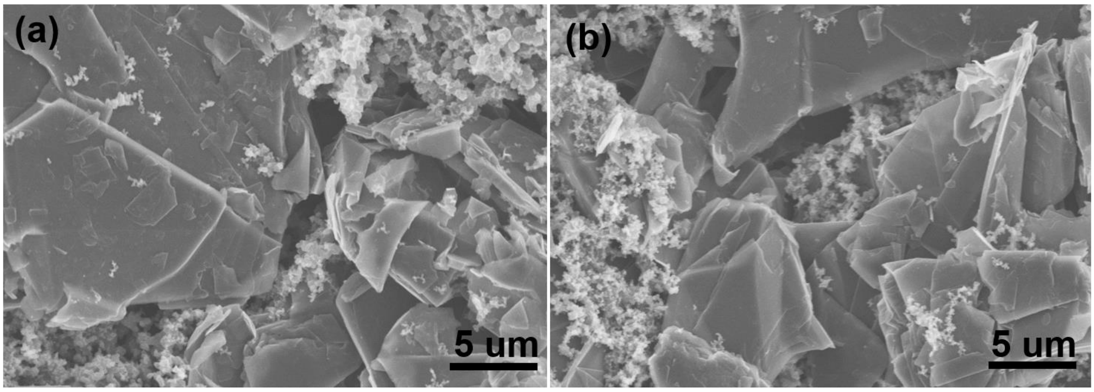

Figure S4. SEM images of the EG cathode before (a) and after 300 cycles (b) in the Ca-DCB.

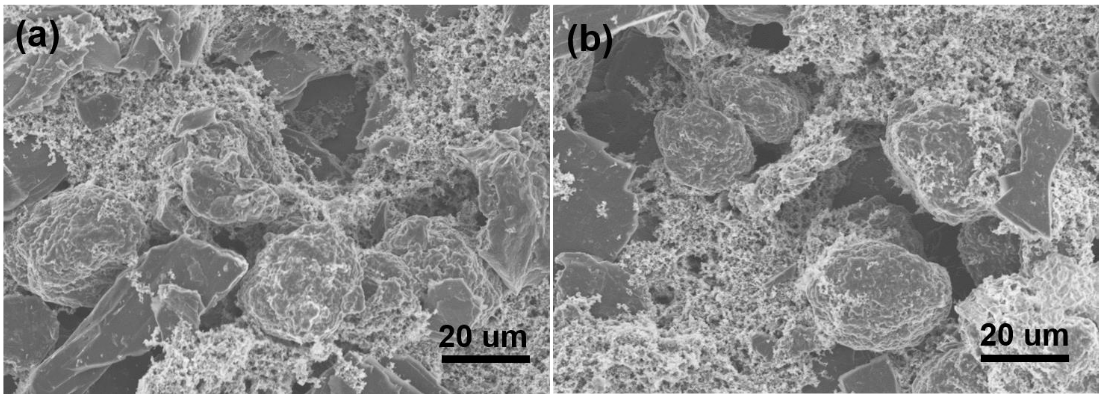

Figure S5. SEM images of the MCMB anode before (a) and after 300 cycles (b) in the Ca-DCB.

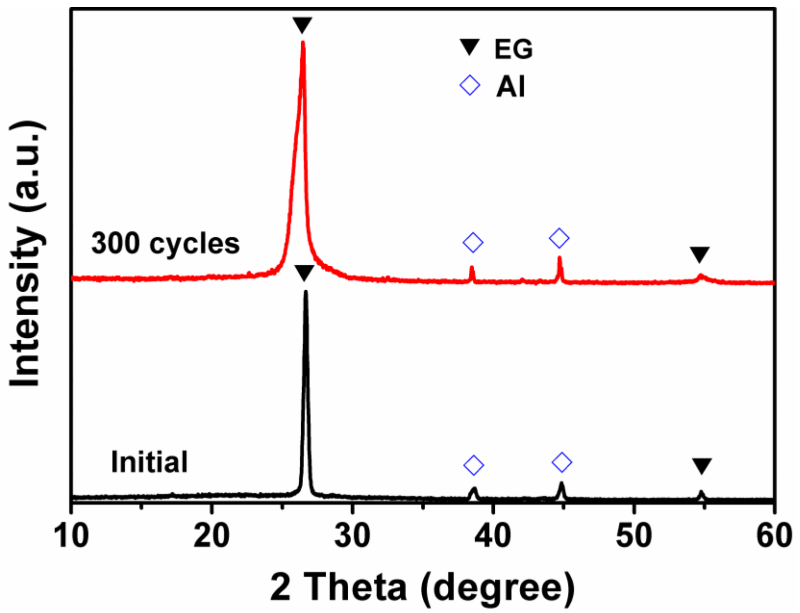

Figure S6. XRD profiles of the EG cathode before and after 300 cycles in the Ca-DCB.

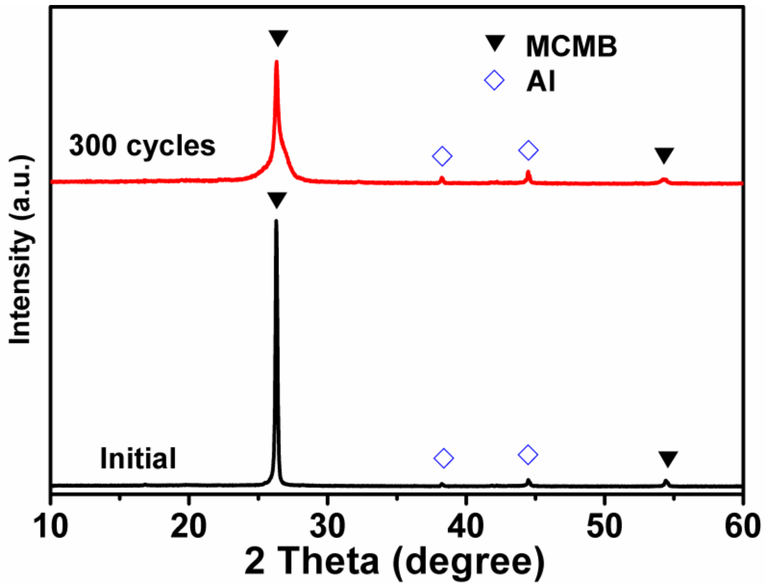

Figure S7. XRD profiles of the MCMB anode before and after 300 cycles in the Ca-DCB.
